# Telomere Attrition in Chronic Kidney Diseases

**DOI:** 10.3390/antiox12030579

**Published:** 2023-02-25

**Authors:** Tina Levstek, Katarina Trebušak Podkrajšek

**Affiliations:** 1 Laboratory for Translational Medical Biochemistry, Institute of Biochemistry and Molecular Genetics, Faculty of Medicine, University of Ljubljana, Vrazov trg 2, 1000 Ljubljana, Slovenia; 2 Clinical Institute for Special Laboratory Diagnostics, University Children’s Hospital, University Medical Centre Ljubljana, Vrazov trg 1, 1000 Ljubljana, Slovenia

**Keywords:** telomere length, chronic kidney disease, oxidative stress, premature ageing, biomarker

## Abstract

Telomeres are dynamic DNA nucleoprotein structures located at the end of chromosomes where they maintain genomic stability. Due to the end replication problem, telomeres shorten with each cell division. Critically short telomeres trigger cellular senescence, which contributes to various degenerative and age-related diseases, including chronic kidney diseases (CKDs). Additionally, other factors such as oxidative stress may also contribute to accelerated telomere shortening. Indeed, telomeres are highly susceptible to oxidative damage due to their high guanine content. Here, we provide a comprehensive review of studies examining telomere length (TL) in CKDs to highlight the association between TL and the development and progression of CKDs in humans. We then focus on studies investigating TL in patients receiving kidney replacement therapy. The mechanisms of the relationship between TL and CKD are not fully understood, but a shorter TL has been associated with decreased kidney function and the progression of nephropathy. Interestingly, telomere lengthening has been observed in some patients in longitudinal studies. Hemodialysis has been shown to accelerate telomere erosion, whereas the uremic milieu is not reversed even in kidney transplantation patients. Overall, this review aims to provide insights into the biological significance of telomere attrition in the pathophysiology of kidney disease, which may contribute to the development of new strategies for the management of patients with CKDs.

## 1. Introduction

**Figure 1:**
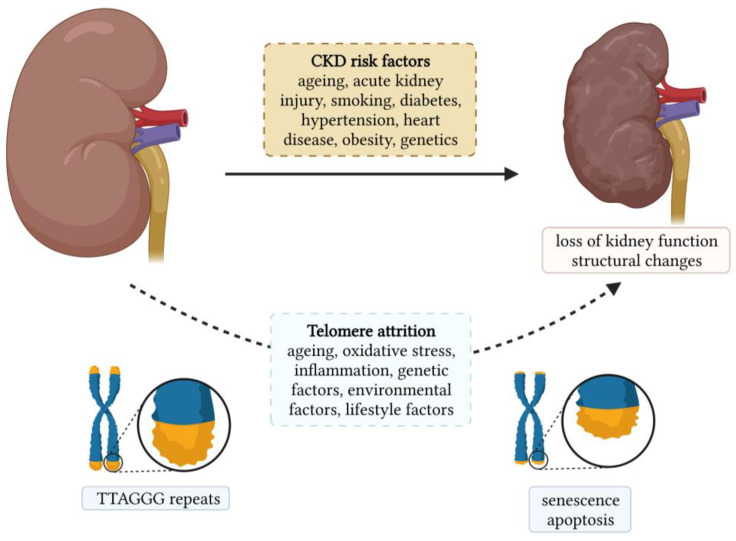
Chronic kidney disease and its association with telomere length.

Chronic kidney diseases (CKDs) are a heterogeneous group of disorders that represent a global public health problem. They affect more than 750 million people worldwide, and this number is expected to increase due to the ageing population [1]. Age-related changes in the kidney are among the most profound among all other organs. Kidney ageing is a complex and multifactorial process in which the kidney undergoes progressive functional and structural changes [2]. Structurally, kidney ageing is characterized by the loss of nephrons and hypertrophy of functional nephrons, glomerulosclerosis, tubular atrophy, interstitial fibrosis, and arteriosclerosis [3]. Functionally, the glomerular filtration rate (GFR) gradually decreases, and the rate of decline increases with age [4]. The changes in tubular function are manifested by a decrease in the ability to conserve and excrete sodium, impaired potassium excretion, and a decreased ability to concentrate and dilute urine [4]. Biological ageing can be assessed by measuring the telomere length (TL), and its role in the development and progression of age-related diseases has been intensively studied in various diseases such as neurodegenerative disorders, cardiovascular diseases, diabetes, monogenic diseases, etc. [5,6,7,8]. The association between CKD and TL is shown in Figure 1, along with the risk factors for CKDs.

Oxidative stress is a hallmark of CKDs and contributes to accelerated telomere attrition. Here, we provide a comprehensive review of studies investigating TL in CKDs and highlight the association between TL shortening and the development and progression of CKDs. We then focus on studies investigating TL in patients who are receiving kidney replacement therapy. Overall, this review provides insights into the biological significance of telomere shortening in the pathophysiology of CKDs, which may contribute to the development of new strategies for managing patients with CKD.

## 2. Telomere Length and the Kidney

Telomeres are nucleoprotein structures that cap the ends of eukaryotic chromosomes. In humans, telomeres consist of non-coding tandem TTAGGG DNA sequences, which are bound by six telomere-specific proteins called shelterin [9]. Telomeres are critical for maintaining genome integrity by preventing DNA damage recognition at the end of chromosomes and the loss of essential genetic information [10]. However, DNA polymerase is unable to fully replicate both DNA strands at the end of linear chromosomes, so telomeres in most somatic cells shorten, by 50–150 base pairs, with each cell division [11]. The length of telomeres is controlled by telomerase, a ribonucleoprotein DNA polymerase complex that catalyzes the addition of telomeric repeats to the 3’ end of the chromosome by reverse transcription of the template region of its associated RNA moiety [12]. However, the expression of this complex is tightly regulated. Telomerase is highly expressed in the early stages of embryonic development and in pluripotent stem cells, while most somatic cells lack telomerase activity [13]. In the absence of telomere elongation, telomeres eventually shorten below a critical length, triggering an activation of the telomeric DNA damage response that leads to replicative senescence or apoptosis [14,15,16]. Senescent cells undergo functional and physiological changes that impair organ function [17].

In the ageing kidney, telomere shortening is faster in the renal cortex than in the medulla and it may contribute to glomerulosclerosis. The differences are greater in younger kidneys and decrease with age [18]. Studies that have investigated the relationship between TL and kidney function in healthy subjects are inconclusive. A study of 139 participants from the Han population (China) reported a significant association between shorter leukocyte TL and lower estimated GFR (eGFR). Participants were between 35 and 90 years of age and were not taking any medication [19]. In contrast, in another cohort that included 471 participants from the Han population with the same age range and inclusion criteria, no association was found between TL shortening and reduced kidney function [20]. However, the subjects were followed for only 3 years, so studies with a longer follow-up are warranted. Another study on the Han population, which included a total of 403 participants with normal and abnormal glucose tolerance, also showed no association between leukocyte TL and kidney function estimated as eGFR or urinary albumin-to-creatinine ratio (UACR) [20,21]. The discrepancies may be partially explained by different markers of kidney function (e.g., eGFR and UACR), different study populations, age-related telomere attrition, and kidney function decline. However, a recent Mendelian randomization study found a casual linkage between genetic predisposition and telomere attrition and a higher risk of CKD, independent of chronological age and comorbidities. The study included 52 independent single nucleotide polymorphisms (SNPs). Conversely, genetically predicted kidney disfunction was associated with accelerated TL shortening [22]. Furthermore, a longitudinal study that included 3964 participants showed that subjects with a longer buccal TL were more likely to maintain normal kidney function. TL, age, sex, race, education, smoking, alcohol consumption, heart disease, diabetes, blood pressure, body mass index, and glycated hemoglobin were significantly associated with the trajectory group [23]. Taken together, these findings indicate the potential role of telomere attrition in altering kidney function. 

## 3. Telomere Length and Chronic Kidney Diseases

The prevalence of CKD is highest in the elderly population. Because TL has been proposed as a marker of biological age, many studies have investigated the association between TL and kidney function, the progression of nephropathy, and the duration of CKDs. These studies are listed in Table 1.

Conflicting results have been published for the relation between TL and kidney function, but most studies reported an association between shorter TL and kidney dysfunction. In the United States National Health and Nutrition Examination Survey, which included more than 10,000 patients, short leukocyte TL was cross-sectionally associated with decreased eGFR and increased UACR [24]. On the other hand, a shorter leukocyte TL was associated with decreased eGFR but not AUCR in Japanese individuals at cardiovascular risk [25]. Shorter TL also correlated with decreased eGFR in patients with chronic heart failure and patients with IgA nephropathy [26,27,28]. In Chinese individuals with type 2 diabetes, the rate of decline in eGFR was linearly related to baseline leukocyte TL [29]. However, in studies concerning patients with a moderate severity of CKD and in patients with coronary heart disease, an age-dependent association between a short TL and reduced eGFR was found [30,31]. In addition, accelerated TL shortening over 5 years was found in patients with higher serum creatinine, cystatin C, and reduced eGFR, with age as a confounding factor, indicating an intertwined relationship between TL shortening and kidney ageing. It is also worth mentioning that the Heart and Soul Study included elderly subjects with a mean age of 66.7 years [31]. A more recent study on patients with heart failure and with a similar median age of 65.9 years found no relationship between TL and kidney function [32]. 

Several studies have demonstrated an association between TL and the progression of CKD. In a prospective study of Chinese patients with type 2 diabetes, a one-unit decrease in leukocyte TL was associated with a 1.2-fold increased risk of end-stage kidney disease (ESKD), independent of the traditional risk factors including age, duration of diabetes, metabolic control, and complications at baseline [29]. In contrast, a study of 157 individuals with type 1 diabetes and impaired kidney function and 116 individuals with normoalbuminuria, followed for an average of 11.1 years, found no association between leukocyte TL and the incidence of ESKD [33]. In the study of two independent cohorts that included patients with mild to moderate CKD, shorter TL at baseline showed a trend toward an increased risk of CKD progression, whereas the effect was significantly modified by smoking and the presence of diabetes [34]. A prospective study of more than 2000 patients with type 2 diabetes showed a 1.9-fold increased risk of albuminuria progression in those with a short leukocyte TL after an adjustment for traditional risk factors compared with those with a longer TL [35]. Similarly, in patients with type 1 diabetes, a shorter leukocyte TL and an increased proportion of short telomeres predicted an increased risk of albuminuria progression. [36]. A shorter TL was also observed in patients with type 2 diabetes and albuminuria compared with normoalbuminuric patients, even after an adjustment for potential confounders [37]. In patients with IgA nephropathy, shorter telomeres were discovered in kidney cells of patients with severe pathological changes and progressive disease compared with patients with normal or mild pathological changes and control subjects. However, this was not true for the progression of diabetic nephropathy, systemic lupus erythematosus, and focal segmental glomerulosclerosis [38]. The role of TL shortening in CKD progression was also demonstrated by Crépin et al., where shorter TLs were found in patients with advanced CKD compared to CKD patients with a normal kidney function [39]. Finally, a recent Mendelian randomization study in an Asian population described an association between genetically determined shorter leukocyte TL and an increased risk of CKD in patients with type 2 diabetes [40]. 

Interesting results were reported in the German Chronic Disease Study, which included 4802 CKD patients. They found a U-shaped relationship between TL and CKD duration. Namely, the shortest telomeres were found in CKD patients with a reported CKD duration of less than 6 months, followed by patients with a CKD duration of more than 5 years. Patients with a duration between 6 months and 5 years had the shortest telomeres [41]. The lengthening of telomeres over time may be explained by increased telomerase activity induced by impaired kidney function or survival bias. Lymphocytes are one of the few mature cells in which telomerase remains active [13]. However, the findings of telomerase activity are inconclusive, as one study found significantly higher telomerase activity in patients with CKD stage five compared to other stages, while telomerase activity was similar in CKD stages two, three, and four. A significant difference was also observed between patients and control subjects [42]. In another study, no difference in telomerase activity was observed between patients with different CKD stages [39].

Patients with CKD have increased morbidity and mortality. Indeed, a shorter TL increased the risk of death in CKD patients [33,39]. Moreover, a German Chronic Kidney Disease Study suggested leukocyte TL as a strong and independent predictor of all-cause mortality during a four-year follow-up of nearly 5000 patients with moderately severe CKD. A 0.1 unit decrease in relative TL was associated with a 14% increased risk of death, independent of age, sex, kidney function, and cardiovascular risk factors. Patients with the shortest TL had a 75% higher risk than patients with the longest TL [43].

**Table 1:** Studies on telomere length in chronic kidney diseases.

Study	Study Population	TL Method	Sample	Main Findings
Cheng et al. (2022) [29]	Patients with type 2 DM	qPCR	Leukocytes	A shorter TL was associated with a higher risk of developing ESKD; a shorter TL at baseline was associated with a faster eGFR decline.
Elena et al. (2021) [32]	HF patients	qPCR	Leukocytes	A shorter TL was not associated with eGFR.
Fazzini et al. (2020) [43]	CKD patients	qPCR	Leukocytes	A shorter TL was associated with all-cause mortality.
Sun et al. (2020) [44]	Patients with primary glomerulonephritis	qPCR	Leukocytes	No difference in TL between patients and control subjects.
Crépin et al. (2020) [39]	CKD patients	qPCR	PBMCs	A shorter TL in patients with advanced CKD compared to patients with normal kidney function; an increased risk of death was observed in patients with shorter TL.
Gurung et al. (2018) [35]	Patients with type 2 DM	qPCR	Leukocytes	A shorter TL was associated with a higher risk for albuminuria progression.
Eguchi et al. (2017) [25]	Subject with at least one CVD risk factor	qPCR	Leukocytes	A shorter TL was associated with reduced eGFR, but not with UACR.
Mazidi et al. (2017) [24]	CKD patients	qPCR	Leukocytes	A shorter TL was associated with reduced eGFR and increased UACR.
Raschenberger et al. (2015) [41]	CKD patients	qPCR	Leukocytes	U-shaped association of TL with CKD duration.
Raschenberger et al. (2015) [30]	CKD patients	qPCR	Leukocytes	Age-dependent association between eGFR and shorter TL.
Raschenberger et al. (2015) [34]	CKD patients	qPCR	Leukocytes	A shorter TL was associated with an increased risk of CKD progression.
Lu et al. (2014) [38]	IgA nephropathy, SLE, DN, and FSGS patients	qFISH	Kidney biopsy	A shorter TL in patients with severe IgA nephropathy was observed compared to control subjects.
Bansal et al. (2012) [31]	CHD patients	qPCR	Leukocytes	Age-dependent association between (1) baseline eGFR and shorter TL and (2) kidney function and faster TL shortening.
Harst et al. (2011) [26]	CHF patients	qPCR	Leukocytes	A shorter TL was associated with reduced eGFR.
Fyhrquist et al. (2010) [36]	Patients with type 1 DM	TRF	Leukocytes	A shorter TL and higher proportion of short telomeres predicted albuminuria progression.
Wong et al. (2009) [27]	Patients with CHF	qPCR	Leukocytes	A shorter TL was associated with reduced eGFR.
Astrup et al. (2010) [33]	Patients with type 1 DM	TRF	Leukocytes	No difference in TL between patients with and without nephropathy; TL was associated with all-cause mortality.
Tentolouris et al. (2007) [37]	Patients with type 2 DM	TRF	Leukocytes	A shorter TL in patients with albuminuria compared to patients without albuminuria.
Szeto et al. (2005) [28]	IgA nephropathy patients	TRF	PBMCs and urinary sediment	A shorter TL of urinary DNA was associated with reduced eGFR but not with glomerulosclerosis and tubulointerstitial scarring; the decrease rate in eGFR was associated with the TL of urinary DNA.

CHD, coronary heart disease; CHF, chronic heart failure; CKD, chronic kidney disease; CVD, cardiovascular disease; DM, diabetes mellitus; DN, diabetic nephropathy; eGFR, estimated glomerular filtration rate; ESKD, end-stage kidney disease; FSGS, focal segmental glomerulosclerosis; HF, heart failure; qFISH, quantitative fluorescence in situ hybridization; qPCR, quantitative polymerase chain reaction; PBMCs, peripheral blood mononuclear cells; SLE, systemic lupus erythematosus; TL, telomere length; TRF, terminal restriction fragment analysis; and UACR, urinary albumin-to-creatinine ratio.

## 4. Telomere Length and Kidney Replacement Therapies

Although premature death is more common in CKD patients than a progression to ESKD [45], the number of patients requiring kidney replacement therapy, such as hemodialysis (HD) or kidney transplantation (KTx), is significant and increasing. Significantly shorter TL of CD4+ T cells have been found in ESKD patients compared to control subjects, regardless of HD [46]. Studies that investigated TL in patients receiving kidney replacement therapy are listed in Table 2.

HD contributes to an inflammatory response beyond that associated with CKD itself [47]. Increased inflammation contributes to accelerated telomere attrition, as several studies have shown a shorter TL or telomeric G-tail in patients on HD compared with control subjects [48,49,50]. Additionally, HD patients treated with active vitamin D had a longer TL than untreated patients [49], which is possibly due to the anti-inflammatory properties of vitamin D. Iron overload, which is common in HD patients and contributes to increased oxidative stress, has been associated with accelerated TL shortening [51]. A short TL has also been associated with inflammatory markers [48,52]. 

**Table 2:** Studies on telomere length in patients on kidney replacement therapy.

Study	Study Population	TL Method	Sample	Main Findings
Wang et al. (2021) [53]	HD and KTx patients	qPCR	Leukocytes	A shorter TL in HD patients without KTx compared to KTx patients.
De Vusser et al. (2020) [54]	KTx patients	qPCR	Kidney biopsy	Intrarenal TL 5 years after Tx was associated with renal arteriosclerosis and reflected kidney donor characteristics.
Kato et al. (2016) [55]	HD patients	qPCR	Leukocytes	Significant TL shortening after one year of HD.
Murillo-Ortiz et al. (2016) [51]	HD patients	qPCR	Leukocytes	TL was inversely related to time on HD.
Luttropp et al. (2016) [56]	HD and KTx patients	qPCR	Leukocytes	Greater telomere attrition in KTx patients compared to HD patients at one year.
De Vusser et al. (2015) [57]	Kidney donors	qPCR	Leukocytes and kidney biopsy	Telomere attrition was associated with the histology of arteriosclerosis.
Stefanidis et al. (2015) [58]	HD patients	TRF	PBMCs	No difference in TL between HD patients and control subjects; a long duration of HD was associated with a shorter TL.
Hirashio et al. (2014) [50]	HD patients	G-tail telomere HPA	PBMCs	Significantly shorter telomeric G-tails in HD patients was observed compared to control subjects; shorter telomeric G-tails were associated with a higher risk of cardiovascular events.
Meijers et al. (2014) [59]	KTx patients	FISH	PBMCs	Significantly shorter TL of T cells in KTx patients compared with control subjects; no difference in TL between pre-RTx and after one-year KTx.
Borras et al. (2012) [49]	HD patients	TRF	PBMCs	A shorter TL in HD patients was observed compared to control subjects.
Carrero et al. (2008) [52]	HD patients	qPCR	Leukocytes	A shorter TL was associated with a higher risk of mortality.
Boxal et al. (2006) [60]	HD patients	TRF	PBMCs	No difference in TL between HD patients and control subjects; TL was negatively associated with time on HD.
Ramírez et al. (2005) [48]	HD patients	FISH	PBMCs	A shorter TL in HD patients was observed compared to control subjects.

FISH, fluorescence in situ hybridization; HD, hemodialysis; HPA, hybridization protection assay; KTx, kidney transplant; qPCR, quantitative polymerase chain reaction; PBMCs, peripheral blood mononuclear cells; TL, telomere length; and TRF, terminal restriction fragment analysis.

TLs of leukocytes and PBMCs were found to be inversely related to the duration of HD [51,53,58,60]. During the first year, patients with longer telomeres showed greater attrition, but some patients were also observed to have prolonged TL, which was associated with an increased leukocyte count at baseline [55]. There are also some conflicting studies that found no difference in TL between HD patients and control subjects [58,60]. As expected, higher telomerase activity in PBMCs was associated with longer TL in HD patients [58]. Telomerase activity in PBMCs was lower in HD patients compared with control subjects and in long-term patients compared with short-term HD patients. However, inflammatory markers were not correlated with telomerase activity [61]. Similar to CKD patients, a shorter TL was associated with an increased mortality risk [52], while a shorter telomeric G-tail was associated with an increased cardiovascular risk [50]. 

KTx patients usually have a better quality of life and better long-term outcomes than HD patients [62]. A recent study showed a shorter TL in HD patients compared with KTx patients [53]. Paradoxically, Luttropp et al. found greater telomere attrition in KTx patients compared with HD patients after one year, but the patients were not age-matched [56]. On the other hand, Meijers et al. showed that KTx did not reverse uremia-associated immunological ageing, as there was no difference in the TL of T cells between pre-KTx and one-year post-KTx [59]. Telomere shortening has also been associated with renal arteriosclerosis [54,57]. Nevertheless, the factors affecting long-term graft longevity remain to be explored. Studies have shown an association between TL and KTx outcome, which was reviewed by Kłoda et al. [63]. 

## 5. Role of Oxidative Stress in Telomere Shortening

Oxidative stress occurs when the formation of reactive oxygen and nitrogen species (RONS) exceeds the endogenous antioxidant capacity [64]. RONS cause damage to various biomolecules, such as proteins, lipids, and nucleic acids. In CKD, impaired mitochondrial function and increased mitochondrial reactive oxygen species (ROS) are major causes of increased oxidative stress [65,66]. It is generally accepted that oxidative stress contributes to the development and progression of CKDs, as described in [67]. 

**Figure 2:**
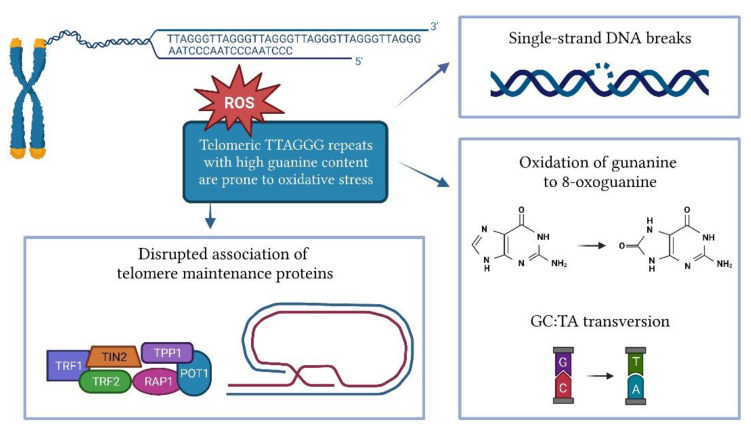
Impact of oxidative stress on telomere length.

Cellular replication is the main cause of telomere shortening, but other factors may also influence the rate of telomere shortening. Several in vitro and in vivo studies have shown that oxidative stress significantly accelerates telomere attrition [68,69]. The shortening of telomeres induced by oxidative stress is tissue-specific and depends on the antioxidant capacity of each tissue [70]. Telomeres are highly susceptible to oxidative damage due to their high content of guanine triples compared to the rest of the genome (Figure 2) [71]. Oxidation of guanine produces 8-oxoguanine (8-oxoG), which is even more susceptible to oxidation, and eventually leads to hydantoin lesions [72]. 8-oxoG is unable to adequately bind to adenine, leading to GC-TA transversion [73]. As a commonly used biomarker of oxidative stress, 8-oxoG can be measured by an enzyme-linked immunosorbent assay (ELISA) or liquid chromatography in various biofluids such as serum, plasma, and urine. Peroxiredoxin (PRDX1), which scavenges hydroxy peroxide, is enriched at the telomeres during replication and counteracts ROS-induced telomeric damage. The loss of PRDX1 results in preferential damage to telomeres [74]. ROS can cause single-strand DNA breaks directly or as an intermediate in lesion repair, leading to replication fork collapse and telomere shortening [75,76]. The damage caused by oxidative stress can also disrupt the association of telomere maintenance proteins such as TRF1, TRF2, and POT1 with DNA [77].

Telomeres are repaired less effectively than the rest of the genome [77]. A study on fibroblasts showed that compared to genomic DNA, where oxidative damage was repaired within 24 h, single-stranded damage in the telomeric region remained unrepaired for at least 19 days [78]. Telomeric DNA can be repaired by base excision repair or, less commonly, by nucleotide extension repair and mismatch repair [79]. Small lesions in DNA are usually repaired by base excision repair [80]. The accumulation of single-strand DNA breaks that occur during base extension repair can lead to replication fork collapse and the formation of double-strand breaks [81]. 8-oxoG is mainly removed by the enzyme 8-oxoguanosine DNA glycolase-1 (OGG1), which can directly remove the 8-oxoG lesion from DNA. However, the efficiency of OGG1 at the 3’-overhang, D-loop, and fork-opening is limited [82].

Long non-coding RNAs, known as telomeric repeat-containing RNAs (TERRA), can be transcribed from subtelomeric regions toward chromosome ends and are actively involved in mechanisms regulating telomere maintenance and chromosome end protection [83]. TERRA can occur as a free nucleoplasmic RNA molecule or as a DNA–RNA hybrid (R-loop), whereby TERRA is associated with telomeric chromatin [84,85]. However, the role of TERRA in telomere shortening is complex and possibly context-dependent. The level of TERRA and TERRA R-loops inversely correspond to the telomere length. In cells with long telomeres, TERRA may act as a negative regulator by binding to telomeric DNA and preventing telomerase access to the telomere [86]. The transcription of TERRA can be induced by telomere dysfunction, which in turn regulates the DNA damage response at dysfunctional telomeres [87]. On the other hand, in critically short telomeres, TERRA and TERRA R-loops are stabilized and promote homology-directed repair and premature senescence [85,88]. Further studies are needed to fully elucidate the mechanisms by which TERRA regulates telomerase activity and telomere maintenance, especially since it has been identified as a potential therapeutic target. Namely, it has been observed that the inhibition of TERRA by antisense oligonucleotides prevents the activation of the DNA damage response and cellular senescence, both in vivo and in vitro [87,89].

## 6. Role of Other Factors in Telomere Shortening

Telomere shortening is a complex process influenced by genetic, environmental, and lifestyle factors, as illustrated in Figure 1. As mentioned earlier, oxidative stress and inflammation are known to contribute toward telomere shortening. Therefore, all factors that increase oxidative stress and/or inflammation result in telomere shortening. Both smoking and obesity are associated with increased oxidative stress and inflammation. A systematic review and meta-analysis showed that TL was shorter in individuals who had ever smoked than in individuals who had never smoked. In addition, TL was shorter in current smokers than in individuals who had quit smoking [90]. Smoking was shown to be associated with a loss of five base pairs of telomeric DNA per year [91]. Obesity is also linked to increased oxidative stress and inflammation. Therefore, it is not surprising that obesity was associated with shorter TL [91]. Interestingly, it has been observed that leukocyte TL can be affected by weight loss. Indeed, greater weight loss was associated with greater telomere lengthening [92]. Healthy dietary patterns such as the Mediterranean diet and the consumption of fruits, vegetables, and antioxidant nutrients have been shown to protect telomeres from shortening, while unhealthy diets such as those rich in processed meat and sweetened beverages contribute toward telomere shortening [93]. The effect of physical activity on TL remains controversial, as about half of the studies found no association between physical activity and TL [94], which is possibly due to differences in frequency and intensity. However, in a cross-sectional study of 5823 adults, longer TLs were found in individuals with high physical activity compared to sedentary individuals. Moreover, the difference was also significant between high and low activity individuals [95]. Nevertheless, moderate activity appears to be sufficient to protect TL, whereas higher activity levels may not provide any additional benefit [96,97]. Sleep quality and duration have also been associated with TL. Poor sleep quality was associated with shorter TL. Moreover, poorer sleep quality predicted shorter TL [98]. Chronic stress exposure was significantly associated with higher oxidative stress, lower telomerase activity, and shorter TL [99]. Overall, healthy lifestyle habits, such as regular exercise, healthy diet, and the avoidance of smoking and daily stress, which lower level of oxidative stress and inflammation, can actively mitigate the ageing process.

In addition to modifiable factors affecting TL, TL has been shown to be a highly heritable trait (36–86%). Several genome-wide association studies have been performed and most identified SNPs harboring genes encoding proteins with known functions in telomere biology [100,101,102,103]. Moreover, the TL has been shown to be longer in adult women [104] and in African Americans compared to Caucasians [105].

## 7. Discussion and Future Perspectives

CKDs are a serious global health problem. As discussed in this review, the accumulating data suggest that telomere shortening is associated with CKDs. Apart from some conflicting results, most studies have shown that telomere shortening is associated with a decline in kidney function and the progression of CKD. During disease progression, TL elongation has been observed in some individuals. HD accelerates TL shortening, whereas KTx is unlikely to reverse the uremic milieu.

Nevertheless, there are still many gaps that need to be addressed in the future. In most studies, TL is measured in blood samples (leukocytes or PBMCs). Studies on kidney samples would be desirable, but a kidney biopsy is an invasive procedure and therefore, it is rarely performed. Although TL varies from tissue to tissue, the Genotype Tissue Expression Project (GTEx), which analyzed *postmortem* tissue samples, has shown that leukocyte TL is a surrogate for TL in the renal cortex [106].

Maintenance of TL is a complex process that is influenced by genetic, epigenetic, and environmental factors. As mentioned above, oxidative stress accelerates the shortening of telomeres [67]. Additionally, some studies have demonstrated that lifestyle changes, diet, and drug interventions can influence TL [107]. Furthermore, age and gender have been reported to influence TL [108]. Because of the various factors that influence telomere shortening, inclusion criteria must be carefully selected to avoid confounding factors that increase telomere attrition. This may be a problem in elderly CKD patients, as they often have several age-associated conditions that may also be involved in the ageing process, such as telomere shortening and senescence [109]. Therefore, the limitations of individual studies have to be critically evaluated when interpreting the results. 

Research designs must be carefully planned and use robust and reproducible methodologies because many technical parameters such as storage conditions and DNA isolation can affect the results [110,111]. The method for TL measurement should be carefully selected according to the purpose of the study, considering its advantages and disadvantages. External and internal controls should be included to ensure reproducibility and the precision of the measurements. Nowadays, qPCR is the most commonly used method for TL measurement. Guidance and recommendations for each step based on the current knowledge have been provided by Lin et al., along with the minimum information needed to characterize the method used, including basic quality metrics that allow comparison of results from different laboratories [111]. The study power may also be an issue in some studies, as often, only a small number of patients are included in the studies, which could lead to statistically non-significant results.

In conclusion, although TL is associated with the development and progression of CKDs, TL alone provides only a crude estimate of the rate of ageing and, thus, can hardly be considered a clinically important biomarker. However, together with biomarkers of oxidative stress and genetic factors, it may help identify patients who are at an increased risk of developing CKD and the rapid progression to kidney failure. In addition, studies on patients with different etiologies of nephropathy are needed to clarify the extent to which telomere shortening contributes to the development and progression of CKDs. Further studies are needed to translate these findings into clinical practice, which could improve the management of CKD patients.

## References

[1] GBD 2015 DALYs, HALE Collaborators (2016). Global, regional, and national disability-adjusted life-years (DALYs) for 315 diseases and injuries and healthy life expectancy (HALE), 1990–2015: A systematic analysis for the Global Burden of Disease Study 2015. Lancet.

[2] Weinstein J.R., Anderson S. (2010). The aging kidney: Physiological changes. Adv. Chronic Kidney Dis..

[3] Lerma E.V. (2009). Anatomic and physiologic changes of the aging kidney. Clin. Geriatr. Med..

[4] Karam Z., Tuazon J. (2013). Anatomic and physiologic changes of the aging kidney. Clin. Geriatr. Med..

[5] Levstek T., Kozjek E., Dolžan V., Trebušak Podkrajšek K. (2020). Telomere attrition in neurodegenerative disorders. Front. Cell. Neurosci..

[6] Deng Y., Li Q., Zhou F., Li G., Liu J., Lv J., Li L., Chang D. (2022). Telomere length and the risk of cardiovascular diseases: A Mendelian randomization study. Front. Cardiovasc. Med..

[7] Tamura Y., Takubo K., Aida J., Araki A., Ito H. (2016). Telomere attrition and diabetes mellitus. Geriatr. Gerontol. Int..

[8] Cokan Vujkovac A., Novaković S., Vujkovac B., Števanec M., Škerl P., Šabovič M. (2020). Aging in Fabry disease: Role of telomere length, telomerase activity, and kidney disease. Nephron.

[9] de Lange T. (2005). Shelterin: The protein complex that shapes and safeguards human telomeres. Genes Dev..

[10] Blackburn E.H. (1984). The molecular structure of centromeres and telomeres. Annu. Rev. Biochem..

[11] Griffith J.D., Comeau L., Rosenfield S., Stansel R.M., Bianchi A., Moss H., de Lange T. (1999). Mammalian telomeres end in a large duplex loop. Cell.

[12] Greider C.W., Blackburn E.H. (1985). Identification of a specific telomere terminal transferase activity in tetrahymena extracts. Cell.

[13] Collins K., Mitchell J.R. (2002). Telomerase in the human organism. Oncogene.

[14] Herbig U., Jobling W.A., Chen B.P.C., Chen D.J., Sedivy J.M. (2004). Telomere shortening triggers senescence of human cells through a pathway involving ATM, p53, and p21(CIP1), but not p16(INK4a). Mol. Cell.

[15] Fumagalli M., Rossiello F., Clerici M., Barozzi S., Cittaro D., Kaplunov J.M., Bucci G., Dobreva M., Matti V., Beausejour C.M. (2012). Telomeric DNA damage is irreparable and causes persistent DNA-damage-response activation. Nat. Cell Biol..

[16] d’Adda di Fagagna F. (2008). Living on a break: Cellular senescence as a DNA-damage response. Nat. Rev. Cancer.

[17] Gonzalez-Gualda E., Baker A.G., Fruk L., Munoz-Espin D. (2021). A guide to assessing cellular senescence in vitro and in vivo. FEBS J..

[18] Melk A., Ramassar V., Lisa M.H.H., Moore R., Rayner D., Solez K., Halloran P.F. (2000). Telomere shortening in kidneys with age. J. Am. Soc. Nephrol..

[19] Zhang W.G., Wang Y., Hou K., Jia L.P., Ma J., Zhao D.L., Zhu S.Y., Bai X.J., Cai G.Y., Wang Y.P. (2015). A correlation study of telomere length in peripheral blood leukocytes and kidney function with age. Mol. Med. Rep..

[20] Zhang W.G., Jia L.P., Ma J., Zhu S.Y., Nie S.S., Song K.K., Liu X.M., Zhang Y.P., Cao D., Yang X.P. (2018). Peripheral blood leukocyte telomere length is associated with age but not renal function: A cross-sectional follow-up study. J. Nutr. Health Aging.

[21] Ma C., He S., Li P., Zhang H., Li W., Li Y. (2020). Negative association between caloric intake and estimated glomerular filtration rate in a Chinese population: Mediation models involving mitochondrial function. Gerontology.

[22] Park S., Lee S., Kim Y., Cho S., Kim K., Kim Y.C., Han S.S., Lee H., Lee J.P., Joo K.W. (2021). A Mendelian randomization study found causal linkage between telomere attrition and chronic kidney disease. Kidney Int..

[23] Westbrook A., Zhang R., Shi M., Razavi A.C., Huang Z., Chen J., He J., Kelly T., Shen Y., Li C. (2022). Association between baseline buccal telomere length and progression of kidney function: The health and retirement study. J. Gerontol. Ser. A.

[24] Mazidi M., Rezaie P., Covic A., Malyszko J., Rysz J., Kengne A.P., Banach M. (2017). Telomere attrition, kidney function, and prevalent chronic kidney disease in the United States. Oncotarget.

[25] Eguchi K., Honig L.S., Lee J.H., Hoshide S., Kario K. (2017). Short telomere length is associated with renal impairment in Japanese subjects with cardiovascular risk. PLoS ONE.

[26] van der Harst P., Wong L.S., de Boer R.A., Brouilette S.W., van der Steege G., Voors A.A., Hall A.S., Samani N.J., Wikstrand J., van Gilst W.H. (2008). Possible association between telomere length and renal dysfunction in patients with chronic heart failure. Am. J. Cardiol..

[27] Wong L.S., van der Harst P., de Boer R.A., Codd V., Huzen J., Samani N.J., Hillege H.L., Voors A.A., van Gilst W.H., Jaarsma T. (2009). Renal dysfunction is associated with shorter telomere length in heart failure. Clin. Res. Cardiol..

[28] Szeto C.C., Poon P.Y.K., Lai F.M.M., Chow K.M., Szeto C.Y.K., Li P.K.T. (2005). Chromosomal telomere shortening of kidney cells in IgA nephropathy by the measurement of DNA in urinary sediment. Clin. Nephrol..

[29] Cheng F., Luk A.O., Wu H., Tam C.H.T., Lim C.K.P., Fan B., Jiang G., Carroll L., Yang A., Lau E.S.H. (2022). Relative leucocyte telomere length is associated with incident end-stage kidney disease and rapid decline of kidney function in type 2 diabetes: Analysis from the Hong Kong Diabetes Register. Diabetologia.

[30] Raschenberger J., Kollerits B., Titze S., Kottgen A., Barthlein B., Ekici A.B., Forer L., Schonherr S., Weissensteiner H., Haun M. (2015). Association of relative telomere length with cardiovascular disease in a large chronic kidney disease cohort: The GCKD study. Atherosclerosis.

[31] Bansal N., Whooley M.A., Regan M., McCulloch C.E., Ix J.H., Epel E., Blackburn E., Lin J., Hsu C.Y. (2012). Association between kidney function and telomere length: The heart and soul study. Am. J. Nephrol..

[32] Elena B., Maria Izarbe M.C., Susana O.G., Sebastian M.G., Jose Luis S.M., Jose Maria D.M., Miguel Angel T.C. (2021). Study of cellular aging in a cohort of patients with heart failure. High Blood Press. Cardiovasc. Prev..

[33] Astrup A.S., Tarnow L., Jorsal A., Lajer M., Nzietchueng R., Benetos A., Rossing P., Parving H.H. (2010). Telomere length predicts all-cause mortality in patients with type 1 diabetes. Diabetologia.

[34] Raschenberger J., Kollerits B., Ritchie J., Lane B., Kalra P.A., Ritz E., Kronenberg F. (2015). Association of relative telomere length with progression of chronic kidney disease in two cohorts: Effect modification by smoking and diabetes. Sci. Rep..

[35] Gurung R.L., Yiamunaa Y., Liu S., Liu J.J., Lim S.C. (2018). Short leukocyte telomere length predicts albuminuria progression in individuals with type 2 diabetes. Kidney Int. Rep..

[36] Fyhrquist F., Tiitu A., Saijonmaa O., Forsblom C., Groop P.H., FinnDiane Study G. (2010). Telomere length and progression of diabetic nephropathy in patients with type 1 diabetes. J. Intern. Med..

[37] Tentolouris N., Nzietchueng R., Cattan V., Poitevin G., Lacolley P., Papazafiropoulou A., Perrea D., Katsilambros N., Benetos A. (2007). White blood cells telomere length is shorter in males with type 2 diabetes and microalbuminuria. Diabetes Care.

[38] Lu Y.Y., Yang X., Chen W.Q., Ju Z.Y., Shou Z.F., Jin J., Zhang X.H., Chen J.H., Jiang H. (2014). Proteins induced by telomere dysfunction are associated with human IgA nephropathy. J. Zhejiang Univ. Sci. B.

[39] Crépin T., Legendre M., Carron C., Vachey C., Courivaud C., Rebibou J.M., Ferrand C., Laheurte C., Vauchy C., Gaiffe E. (2020). Uraemia-induced immune senescence and clinical outcomes in chronic kidney disease patients. Nephrol. Dial. Transplant..

[40] Gurung R.L., Dorajoo R., Wang L., Liu S., Liu J.J., Shao Y.M., Chen Y., Sim X., Ang K., Subramaniam T. (2021). Association of leukocyte telomere length with chronic kidney disease in East Asians with type 2 diabetes: A Mendelian randomization study. Clin. Kidney J..

[41] Raschenberger J., Kollerits B., Titze S., Kottgen A., Barthlein B., Ekici A.B., Forer L., Schonherr S., Weissensteiner H., Haun M. (2015). Do telomeres have a higher plasticity than thought? Results from the German Chronic Kidney Disease (GCKD) study as a high-risk population. Exp. Gerontol..

[42] Kidir V., Aynali A., Altuntas A., Inal S., Aridogan B., Sezer M.T. (2017). Telomerase activity in patients with stage 2–5D chronic kidney disease. Nefrología.

[43] Fazzini F., Lamina C., Raschenberger J., Schultheiss U.T., Kotsis F., Schonherr S., Weissensteiner H., Forer L., Steinbrenner I., Meiselbach H. (2020). Results from the German Chronic Kidney Disease (GCKD) study support association of relative telomere length with mortality in a large cohort of patients with moderate chronic kidney disease. Kidney Int..

[44] Sun Q., Liu J., Cheng G., Dai M., Liu J., Qi Z., Zhao J., Li W., Kong F., Liu G. (2020). The telomerase gene polymorphisms, but not telomere length, increase susceptibility to primary glomerulonephritis/end stage renal diseases in females. J. Transl. Med..

[45] Chronic Kidney Disease Prognosis C., Matsushita K., van der Velde M., Astor B.C., Woodward M., Levey A.S., de Jong P.E., Coresh J., Gansevoort R.T. (2010). Association of estimated glomerular filtration rate and albuminuria with all-cause and cardiovascular mortality in general population cohorts: A collaborative meta-analysis. Lancet.

[46] Betjes M.G., Langerak A.W., van der Spek A., de Wit E.A., Litjens N.H. (2011). Premature aging of circulating T cells in patients with end-stage renal disease. Kidney Int..

[47] Jofre R., Rodriguez-Benitez P., Lopez-Gomez J.M., Perez-Garcia R. (2006). Inflammatory syndrome in patients on hemodialysis. J. Am. Soc. Nephrol..

[48] Ramírez R., Carracedo J., Soriano S., Jimenez R., Martin-Malo A., Rodriguez M., Blasco M., Aljama P. (2005). Stress-induced premature senescence in mononuclear cells from patients on long-term hemodialysis. Am. J. Kidney Dis..

[49] Borras M., Panizo S., Sarro F., Valdivielso J.M., Fernandez E. (2012). Assessment of the potential role of active vitamin D treatment in telomere length: A case-control study in hemodialysis patients. Clin. Ther..

[50] Hirashio S., Nakashima A., Doi S., Anno K., Aoki E., Shimamoto A., Yorioka N., Kohno N., Masaki T., Tahara H. (2014). Telomeric G-tail length and hospitalization for cardiovascular events in hemodialysis patients. Clin. J. Am. Soc. Nephrol..

[51] Murillo-Ortiz B., Ramirez Emiliano J., Hernandez Vazquez W.I., Martinez-Garza S., Solorio-Meza S., Albarran-Tamayo F., Ramos-Rodriguez E., Benitez-Bribiesca L. (2016). Impact of oxidative stress in premature aging and iron overload in hemodialysis patients. Oxid. Med. Cell. Longev..

[52] Carrero J.J., Stenvinkel P., Fellstrom B., Qureshi A.R., Lamb K., Heimburger O., Barany P., Radhakrishnan K., Lindholm B., Soveri I. (2008). Telomere attrition is associated with inflammation, low fetuin-A levels and high mortality in prevalent haemodialysis patients. J. Intern. Med..

[53] Wang Y., Chen S., Feng S., Wang C., Jiang H., Rong S., Hermann H., Chen J., Zhang P. (2021). Telomere shortening in patients on long-term hemodialysis. Chronic Dis. Transl. Med..

[54] De Vusser K., Martens D., Lerut E., Kuypers D., Nawrot T.S., Naesens M. (2020). Replicative senescence and arteriosclerosis after kidney transplantation. Nephrol. Dial. Transplant..

[55] Kato S., Shiels P.G., McGuinness D., Lindholm B., Stenvinkel P., Nordfors L., Qureshi A.R., Yuzawa Y., Matsuo S., Maruyama S. (2016). Telomere attrition and elongation after chronic dialysis initiation in patients with end-stage renal disease. Blood Purif..

[56] Luttropp K., Nordfors L., McGuinness D., Wennberg L., Curley H., Quasim T., Genberg H., Sandberg J., Sonnerborg I., Schalling M. (2016). Increased telomere attrition after renal transplantation-Impact of antimetabolite therapy. Transpl. Direct..

[57] De Vusser K., Pieters N., Janssen B., Lerut E., Kuypers D., Jochmans I., Monbaliu D., Pirenne J., Nawrot T., Naesens M. (2015). Telomere length, cardiovascular risk and arteriosclerosis in human kidneys an observational. Aging.

[58] Stefanidis I., Voliotis G., Papanikolaou V., Chronopoulou I., Eleftheriadis T., Kowald A., Zintzaras E., Tsezou A. (2015). Telomere length in peripheral blood mononuclear cells of patients on chronic hemodialysis is related with telomerase activity and treatment duration. Artif. Organs.

[59] Meijers R.W., Litjens N.H., de Wit E.A., Langerak A.W., Baan C.C., Betjes M.G. (2014). Uremia-associated immunological aging is stably imprinted in the T-cell system and not reversed by kidney transplantation. Transpl. Int..

[60] Boxall M.C., Goodship T.H., Brown A.L., Ward M.C., von Zglinicki T. (2006). Telomere shortening and haemodialysis. Blood Purif..

[61] Tsirpanlis G., Chatzipanagiotou S., Boufidou F., Kordinas V., Alevyzaki F., Zoga M., Kyritsis I., Stamatelou K., Triantafyllis G., Nicolaou C. (2006). Telomerase activity is decreased in peripheral blood mononuclear cells of hemodialysis patients. Am. J. Nephrol..

[62] Tonelli M., Wiebe N., Knoll G., Bello A., Browne S., Jadhav D., Klarenbach S., Gill J. (2011). Systematic review: Kidney transplantation compared with dialysis in clinically relevant outcomes. Am. J. Transpl..

[63] Kłoda K., Domański L., Mierzecki A. (2017). Telomere length assessment for prediction of organ transplantation outcome. Future or failure: A review of the literature. Med. Sci. Monit..

[64] Sies H. (1997). Oxidative stress: Oxidants and antioxidants. Exp. Physiol..

[65] Locatelli F., Canaud B., Eckardt K.U., Stenvinkel P., Wanner C., Zoccali C. (2003). Oxidative stress in end-stage renal disease: An emerging threat to patient outcome. Nephrol. Dial. Transplant..

[66] Galvan D.L., Green N.H., Danesh F.R. (2017). The hallmarks of mitochondrial dysfunction in chronic kidney disease. Kidney Int..

[67] Daenen K., Andries A., Mekahli D., Van Schepdael A., Jouret F., Bammens B. (2019). Oxidative stress in chronic kidney disease. Pediatr. Nephrol..

[68] Reichert S., Stier A. (2017). Does oxidative stress shorten telomeres in vivo? A review. Biol. Lett..

[69] Barnes R.P., Fouquerel E., Opresko P.L. (2019). The impact of oxidative DNA damage and stress on telomere homeostasis. Mech. Ageing Dev..

[70] Cattan V., Mercier N., Gardner J.P., Regnault V., Labat C., Maki-Jouppila J., Nzietchueng R., Benetos A., Kimura M., Aviv A. (2008). Chronic oxidative stress induces a tissue-specific reduction in telomere length in CAST/Ei mice. Free Radic. Biol. Med..

[71] Kawanishi S., Oikawa S. (2004). Mechanism of telomere shortening by oxidative stress. Ann. N. Y. Acad. Sci..

[72] Fleming A.M., Burrows C.J. (2017). Formation and processing of DNA damage substrates for the hNEIL enzymes. Free Radic. Biol. Med..

[73] von Zglinicki T., Martin-Ruiz C.M., Saretzki G. (2005). Telomeres, cell senescence and human ageing. Signal Transduct..

[74] Aeby E., Ahmed W., Redon S., Simanis V., Lingner J. (2016). Peroxiredoxin 1 protects telomeres from oxidative damage and preserves telomeric DNA for extension by telomerase. Cell Rep..

[75] von Zglinicki T., Pilger R., Sitte N. (2000). Accumulation of single-strand breaks is the major cause of telomere shortening in human fibroblasts. Free Radic. Biol. Med..

[76] von Zglinicki T. (2002). Oxidative stress shortens telomeres. Trends Biochem. Sci..

[77] Opresko P.L., Fan J., Danzy S., Wilson D.M., Bohr V.A. (2005). Oxidative damage in telomeric DNA disrupts recognition by TRF1 and TRF2. Nucleic Acids Res..

[78] Petersen S., Saretzki G., von Zglinicki T. (1998). Preferential accumulation of single-stranded regions in telomeres of human fibroblasts. Exp. Cell Res..

[79] Shafirovich V., Geacintov N.E. (2017). Removal of oxidatively generated DNA damage by overlapping repair pathways. Free Radic. Biol. Med..

[80] Cadet J., Davies K.J.A. (2017). Oxidative DNA damage & repair: An introduction. Free Radic. Biol. Med..

[81] Sobol R.W., Prasad R., Evenski A., Baker A., Yang X.P., Horton J.K., Wilson S.H. (2000). The lyase activity of the DNA repair protein β-polymerase protects from DNA-damage-induced cytotoxicity. Nature.

[82] Rhee D.B., Ghosh A., Lu J., Bohr V.A., Liu Y. (2011). Factors that influence telomeric oxidative base damage and repair by DNA glycosylase OGG1. DNA Repair.

[83] Bettin N., Oss Pegorar C., Cusanelli E. (2019). The emerging roles of TERRA in telomere maintenance and genome stability. Cells.

[84] Pfeiffer V., Crittin J., Grolimund L., Lingner J. (2013). The THO complex component Thp2 counteracts telomeric R-loops and telomere shortening. EMBO J..

[85] Balk B., Dees M., Bender K., Luke B. (2014). The differential processing of telomeres in response to increased telomeric transcription and RNA-DNA hybrid accumulation. RNA Biol..

[86] Maicher A., Kastner L., Luke B. (2012). Telomeres and disease: Enter TERRA. RNA Biol..

[87] Aguado J., Sola-Carvajal A., Cancila V., Revechon G., Ong P.F., Jones-Weinert C.W., Wallen Arzt E., Lattanzi G., Dreesen O., Tripodo C. (2019). Inhibition of DNA damage response at telomeres improves the detrimental phenotypes of Hutchinson-Gilford Progeria Syndrome. Nat. Commun..

[88] Graf M., Bonetti D., Lockhart A., Serhal K., Kellner V., Maicher A., Jolivet P., Teixeira M.T., Luke B. (2017). Telomere length determines TERRA and R-Loop regulation through the cell cycle. Cell.

[89] Rossiello F., Aguado J., Sepe S., Iannelli F., Nguyen Q., Pitchiaya S., Carninci P., d’Adda di Fagagna F. (2017). DNA damage response inhibition at dysfunctional telomeres by modulation of telomeric DNA damage response RNAs. Nat. Commun..

[90] Astuti Y., Wardhana A., Watkins J., Wulaningsih W., Network P.R. (2017). Cigarette smoking and telomere length: A systematic review of 84 studies and meta-analysis. Environ. Res..

[91] Valdes A.M., Andrew T., Gardner J.P., Kimura M., Oelsner E., Cherkas L.F., Aviv A., Spector T.D. (2005). Obesity, cigarette smoking, and telomere length in women. Lancet.

[92] Carulli L., Anzivino C., Baldelli E., Zenobii M.F., Rocchi M.B., Bertolotti M. (2016). Telomere length elongation after weight loss intervention in obese adults. Mol. Genet. Metab..

[93] Galie S., Canudas S., Muralidharan J., Garcia-Gavilan J., Bullo M., Salas-Salvado J. (2020). Impact of nutrition on telomere health: Systematic review of observational cohort studies and randomized clinical trials. Adv. Nutr..

[94] Chilton W., O’Brien B., Charchar F. (2017). Telomeres, aging and exercise: Guilty by association?. Int. J. Mol. Sci..

[95] Tucker L.A. (2017). Physical activity and telomere length in U.S. men and women: An NHANES investigation. Prev. Med..

[96] Denham J., O’Brien B.J., Prestes P.R., Brown N.J., Charchar F.J. (2016). Increased expression of telomere-regulating genes in endurance athletes with long leukocyte telomeres. J. Appl. Physiol..

[97] Ludlow A.T., Zimmerman J.B., Witkowski S., Hearn J.W., Hatfield B.D., Roth S.M. (2008). Relationship between physical activity level, telomere length, and telomerase activity. Med. Sci. Sports Exerc..

[98] Prather A.A., Puterman E., Lin J., O’Donovan A., Krauss J., Tomiyama A.J., Epel E.S., Blackburn E.H. (2011). Shorter leukocyte telomere length in midlife women with poor sleep quality. J. Aging Res..

[99] Epel E.S., Blackburn E.H., Lin J., Dhabhar F.S., Adler N.E., Morrow J.D., Cawthon R.M. (2004). Accelerated telomere shortening in response to life stress. Proc. Natl. Acad. Sci. USA.

[100] Njajou O.T., Cawthon R.M., Damcott C.M., Wu S.-H., Ott S., Garant M.J., Blackburn E.H.M., Braxton D., Shuldiner A.R., Hsueh W.-C. (2007). Telomere length is paternally inherited and is associated with parental lifespan. Proc. Natl. Acad. Sci. USA.

[101] Andrew T., Aviv A., Falchi M., Surdulescu G.L., Gardner J.P., Lu X., Kimura M., Kato B.S., Valdes A.M., Spector T.D. (2006). Mapping genetic loci that determine leukocyte telomere length in a large sample of unselected female sibling pairs. Am. J. Hum. Genet..

[102] Vasa-Nicotera M., Brouilette S., Mangino M., Thompson J.R., Braund P., Clemitson J.R., Mason A., Bodycote C.L., Raleigh S.M., Louis E. (2005). Mapping of a major locus that determines telomere length in humans. Am. J. Hum. Genet..

[103] Codd V., Nelson C.P., Albrecht E., Mangino M., Deelen J., Buxton J.L., Hottenga J.J., Fischer K., Esko T., Surakka I. (2013). Identification of seven loci affecting mean telomere length and their association with disease. Nat. Genet..

[104] Benetos A., Okuda K., Lajemi M., Kimura M., Thomas F., Skurnick J., Labat C., Bean K., Aviv A. (2001). Telomere length as an indicator of biological aging: The gender effect and relation with pulse pressure and pulse wave velocity. Hypertension.

[105] Hunt S.C., Chen W., Gardner J.P., Kimura M., Srinivasan S.R., Eckfeldt J.H., Berenson G.S., Aviv A. (2008). Leukocyte telomeres are longer in African Americans than in whites: The National Heart, Lung, and Blood Institute Family Heart Study and the Bogalusa Heart Study. Aging Cell.

[106] Demanelis K., Jasmine F., Chen L.S., Chernoff M., Tong L., Delgado D., Zhang C., Shinkle J., Sabarinathan M., Lin H. (2020). Determinants of telomere length across human tissues. Science.

[107] Navarro-Ibarra M.J., Hernández J., Caire-Juvera G. (2019). Diet, physical activity and telomere length in adults. Nutr. Hosp..

[108] Cherif H., Tarry J.L., Ozanne S.E., Hales C.N. (2003). Ageing and telomeres: A study into organ- and gender-specific telomere shortening. Nucleic Acids Res..

[109] Ortiz A., Mattace-Raso F., Soler M.J., Fouque D. (2022). Ageing meets kidney disease. Age Ageing.

[110] Cunningham J.M., Johnson R.A., Litzelman K., Skinner H.G., Seo S., Engelman C.D., Vanderboom R.J., Kimmel G.W., Gangnon R.E., Riegert-Johnson D.L. (2013). Telomere length varies by DNA extraction method: Implications for epidemiologic research. Cancer Epidemiol. Biomark. Prev..

[111] Lin J., Smith D.L., Esteves K., Drury S. (2019). Telomere length measurement by Qpcr–Summary of critical factors and recommendations for assay design. Psychoneuroendocrinology.

